# Characterization of ^241^Pu occurrence, distribution, and bioaccumulation in seabirds from northern Eurasia

**DOI:** 10.1007/s11356-014-3975-4

**Published:** 2014-12-23

**Authors:** Dagmara I. Strumińska-Parulska, Bogdan Skwarzec

**Affiliations:** Faculty of Chemistry, Analytics and Environmental Radiochemistry Chair, University of Gdańsk, Wita Stwosza 63, 80-308 Gdańsk, Poland

**Keywords:** Plutonium ^241^Pu, ^241^Pu/^239+240^Pu activity ratio, Seabirds, Baltic Sea, Eurasia, Bioaccumulation

## Abstract

The paper presents unique data of plutonium ^241^Pu study in seabirds from northern Eurasia, permanently or temporally living at the southern Baltic Sea coast. Together, ten marine birds species were examined, as follows: three species that permanently reside at the southern Baltic, four species of wintering birds, and three species of migrating birds; 366 samples were analyzed. The obtained results indicated plutonium was non-uniformly distributed in organs and tissues of analyzed seabirds. The highest ^241^Pu content was found in the digestion organs and feathers, the lowest in muscles. Also, the internal radiation doses from ^241^Pu were evaluated.

## Introduction

Among radionuclides in the environment, artificial radioisotopes play a significant role in the toxic effects connected with its accumulation in organisms. Man-made plutonium is widespread all over the world, takes part in geochemical circulation and accumulates in the food chain (Aarkrog 1977; Burger et al. 2007; Strumińska-Parulska and Skwarzec 2012). Plutonium is by far the most important transuranic element, and its isotopes generally found in the environment are ^238^Pu, ^239^Pu, ^240^Pu, and ^241^Pu (Skwarzec 1995; Donard et al. 2007). Plutonium is present in the environment as a consequence of atmospheric weapon testing, reprocessing of nuclear fuel (e.g., Sellafield, Cap the la Hague, Mayak) and accidents in nuclear facilities (as the Chernobyl accident) (Aarkrog 1991; Skwarzec 1995; Varga and Tarjan 2008; Bisinger et al. 2010). Due to the lack of stable plutonium isotopes and their long half-lives, plutonium is considered one of the most important radioactive elements in safety assessment of environmental radioactivity and nuclear waste management (Burger et al. 2007). Plutonium is a radioactive and one of the most toxic metals, and it is a hazardous environmental pollutant. There are two aspects to the harmful effects of plutonium, including radioactivity and heavy metal poison effects (Heiserman 1997). Plutonium is considered a “boneseeker” and accumulates in liver as well (Mietelski et al. 2008). Moreover, ^241^Pu (*T*_1/2_ = 14.35 years) decays by β-emission to the long-lived, highly radiotoxic α-emitting ^241^Am (*T*_1/2_ = 432.2 years), which has health hazards even in small concentrations due to its extremely high radiotoxicity (Mussalo et al. 1980; Hoffmann 2002; Strumińska and Skwarzec 2006). Most of all studies on plutonium have focused on alpha-emitting isotopes so far, namely ^238^Pu, ^239^Pu, and ^240^Pu. There are not many data available concerning the activity concentrations of β-emitting ^241^Pu in biological samples. ^241^Pu seems to be less important in terms of its radiotoxicity than the α-emitting plutonium radionuclides (^238,239,240^Pu) but is quite significant because of its substantial contribution to the whole plutonium fallout.

Besides, the ^241^Pu/^239+240^Pu activity ratio is a fingerprint which reveals the origin of plutonium contamination. For example, nuclear weapon tests fallout was characterized by a ^241^Pu/^239+240^Pu activity ratio of about 12–16 for the latitudes 40–50° North in 1963; for the Chernobyl accident deposition, the ^241^Pu/^239+240^Pu activity ratio was higher, reaching value of around 55–90 in 1986 (Perkins and Thomas 1980; Holm et al. 1992; Irlweck and Wicke 1998; Salminen-Paatero et al. 2014). Nuclear weapon-grade plutonium, which was another important source of this element in the environment, was characterized by a much lower ^241^Pu/^239+240^Pu activity ratio of about 0.5–4 (Irlweck and Hrnecek 1999; Salminen and Paatero 2009; Salminen-Paatero et al. 2014). This is why the simultaneous determination of ^241^Pu and the alpha emitting radioisotopes could be a useful tool for tracing plutonium sources in the environment (Corcho Alvarado et al. 2011).

There is a huge lack of data on ^241^Pu distribution and accumulation in animals. Most of the studies on ^241^Pu distribution in a large extent referred to soils and its local contamination, like Palomares (Spain) (Gasco et al. 1997) and Sellafield (UK) (Moreno et al. 1998; Merino et al. 2000); post-Chernobyl-accident research in Ukraine (Buzinny et al. 1994), Finland (Paatero and Jaakkola 1994; Ikähaimonen 2000), and Poland (Mietelski et al. 1999; Komosa and Piekarz 2009); and our studies referred to the Baltic Sea (Strumińska and Skwarzec 2006; Strumińska-Parulska and Skwarzec 2013). Our previous experiments on the air dust samples collected in 1986 over Gdynia (northern Poland), the year of the Chernobyl accident, indicated extreme increase of ^241^Pu amount in the atmospheric dust; in April 1986, the ^241^Pu activity reached 3643 Bq kg^−1^ dry weight (dw). Starting from May 1986 (33.1 Bq kg^−1^ dw), the ^241^Pu concentrations in the air dust were decreasing systematically, and in November 1986, it reached the level before the Chernobyl accident (1.08 Bq kg^−1^ dw). The ^241^Pu/^239+240^Pu activity ratio in the air dust samples increased from 34 (March 1986) to 56 (April 1986) after the accident and decreased slowly reaching 36 (December 1986) (Strumińska and Skwarzec 2006). Similar situations were observed in Vienna and Salzburg (Austria), Belgrade (Serbia), and Vilnius (Lithuania) (Irlweck and Wicke 1998; Vukanac et al. 2006; Lujaniene et al. 2009). Mietelski et al. (1999) suggested that the initial (at the moment of the Chernobyl accident) deposition of ^241^Pu in Poland might have been relatively high, up to the level of 2 kBq m^−2^. The maximum result of ^241^Pu in forest soil was estimated at 254 Bq kg^−1^ dw, and the enhanced levels of this isotope were observed in all samples from the north-eastern Poland. Our previous researches showed that the principal source of ^241^Pu on the Polish territory and the southern Baltic area was the Chernobyl accident (Strumińska and Skwarzec 2006; Strumińska-Parulska and Skwarzec 2012; Strumińska-Parulska and Skwarzec 2013).

Plutonium can be accumulated in the biota, and for this reason, it could be an important source of radiation dose in the body of animals (Skwarzec 1995; Chibowski et al. 2006; Mietelski et al. 2008). Birds are characterized by high body temperature and intensive metabolism what cause high daily food requirement (Tomiałojć and Stawarczyk 2003). Most of them are multi-habitat species, and they are a significant part of the biotas. Seabirds are a very important element of the trophic chain of marine ecosystem as well. Particularly, the birds’ feathers are often used as bioindicators of heavy metals contamination in marine and air environment (Pilastro et al. 1993; Burger and Gochfeld 1997). There were high differences observed in some radionuclide concentrations among migratory and sedentary birds. However, migratory birds, as it appears, cumulate more radionuclides, as they operate in many, diversified habitats (Krumholz 1954; Krivolutsky et al. 1999; Mietelski et al. 2006; Burger et al. 2007; Kitowski et al. 2008; Mietelski et al. 2008; Gaschak et al. 2009; Kitowski et al. 2009; Howard et al. 2013). Matishov et al. (1996) reported on caesium-137 in seabirds in the Barents Sea, but very few data on radionuclide levels in seabirds are available. One might anticipate that levels in mollusc-eating shorebirds and seaducks could be elevated in areas such as the Cumbrian coast, but this does not seem to have been investigated (Tasker and Furness 2003).

From over 9000 species of birds in the world, approximately 200 species are related to the Polish part of the Baltic coastal zone (Żmudziński 1990; Tomiałojć and Stawarczyk 2003). That is why they could be one of the constituent in radionuclides transport (Navarro et al. 1998). Brisbin (1993) stated seabirds were probably not very useful in radionuclide monitoring because levels did not tend to increase food chains and the assimilation efficiency of most radionuclides through the digestive system of seabirds was poor. However, the knowledge on ^241^Pu distribution is indispensable for the correct assessment of its radioactive contamination and the radiological consequences. Current knowledge about bioaccumulation of plutonium ^241^Pu in birds is still very poor. In Eurasia, there were no studies on ^241^Pu contamination in birds (Krivolutsky et al. 1999; Burger et al. 2007; Kitowski et al. 2008; Mietelski et al. 2008; Gaschak et al. 2009). While models are useful in predicting what concentrations might be expected in different biota compartments in ecosystems, measurements of actual concentrations in biota and food consumed would be clearly more directly useful in predicting intake rates and ultimately doses (Burger et al. 2007).

The paper presents the results of ^241^Pu activity concentration measurements in tissues and organs of ten seabird species collected at the southern Baltic Sea coast. The aim of the investigation was to assess the level of ^241^Pu contamination of marine birds that live in northern Eurasia, indicate the main bioaccumulation organs, calculate the values of bioconcentration factors (BCFs), and evaluate the internal radiation doses from ^241^Pu. Moreover, the present study can help to recognize the plutonium sources in marine birds, link the diet and living habits to the differences in plutonium distribution, and provide valuable information about plutonium transfer between atmosphere and sea.

## Materials and methods

Among bird species registered at the southern Baltic Sea coast, there are main groups that permanently reside (principally at the Gdańsk Bay and the Puck Bay) as well as wintering and migratory birds. The following ten species of seabirds breeding or living in northern Eurasia were collected for research:

1. Seabirds that permanently reside at the southern Baltic Sea: tufted duck (*Aythya fuligula*), Eurasian coot (*Fulica atra*), and great cormorant (*Phalacrocorax carbo*);
2. Wintering birds: common eider (*Somateria mollissima*), velvet scoter (*Melanitta fusca*), black guillemot (*Cepphus grylle*), and long-tailed duck (*Clangula hyemalis*);
3. Migratory birds: razorbill (*Alca torda*), common guillemot (*Uria aalge*), and red-throated diver (*Gavia stellata*).

**Fig. 1:**
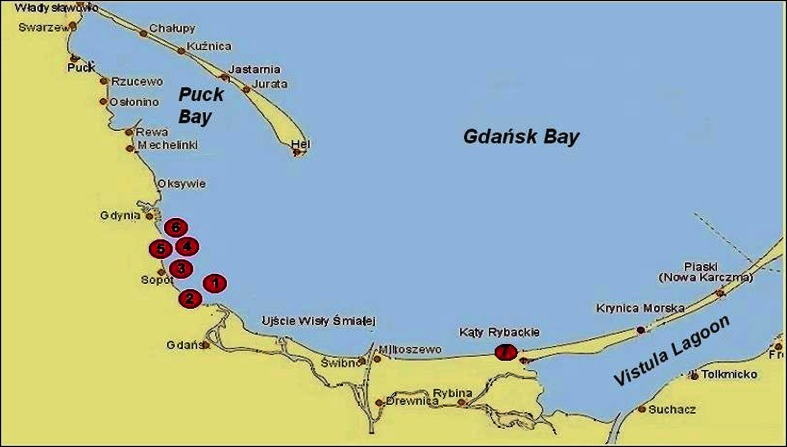
Locations of sampling sites: (1) 2–3 miles from the Jelitkowo beach; (2) Jelitkowo beach (Gdańsk); (3) 1.5 miles from Sopot beach; (4) 1–2 miles from Orłowo beach; (5) Orłowo beach (Gdynia); (6) 1–4 miles from Gdynia city beach; and (7) Kąty Rybackie beach

The analyzed material contained dead marine birds found on the beach or caught by fishermen while fishing in 2003–2005. The locations of sampling sites are presented in Fig. 1. All analyzed birds were adult—over 3 years old. In the laboratory, the birds were weighted and the following organs and tissues were dissected, giving 366 subsamples of feathers, skin, muscles, liver, skeleton, and viscera (internal organs and the rest). Because of small plutonium activities expected, all collected materials were used. The fresh samples were weighted, and their masses were from 50 to 1000 g, then homogenized (Thermomix, Vorwerk, Germany) and digested using 65 % HNO_3_ with a ^242^Pu (5 mBq) spikes added as a yield tracers before the radiochemical analysis. The plutonium analysis was treated as follows: samples mineralization in nitric acid, separation and purification on ion resins, and electrolysis on a steel disc of all plutonium isotopes. The specific electrolysis conditions allow for plutonium deposition only (Skwarzec 1995, 1997; Skwarzec 2010). At first, the activities of ^238^Pu and ^239+240^Pu radionuclides were measured in alpha spectrometer (Strumińska-Parulska et al. 2011). The alpha plutonium samples were measured at latest 1 month after electrolysis. This time is sufficient to avoid significant ^241^Am ingrowth from ^241^Pu decay and, as calculated, the maximum decrease in its activity could be 0.004 %. The minimum detectable activity (MDA) for ^239 + 240^Pu was 0.05 mBq. Furthermore, the indirect determination of ^241^Pu was done by measuring the increment in ^241^Am from the decay of β-emitting ^241^Pu, and all alpha spectrometric sources were remeasured using the alpha spectrometer Canberra Packard Alpha Analyst equipped with 12 PIPS detectors (300 and 450 mm^2^ area each, FWHM = 17–18 keV). Each sample was measured about 1 month. The alpha plutonium spectra acquired were compared with the respective spectra obtained 5–10 years earlier (Strumińska-Parulska et al. 2011). A comparison of the obtained spectra allowed for the estimation of the ^241^Pu content based on the increment of the 5.49-MeV peak of ^241^Am. ^238^Pu previously present in the samples, mainly from the Chernobyl accident, and its decay were taken into account during the calculation. All ^241^Pu activity concentrations were calculated on the sampling time. The calculation of the ^241^Pu activity was based on the following formula (1):

1 $$ {A_{\mathrm{Pu}}}_{{}_0}=30.11409\cdot \frac{A_{{}^{241}\mathrm{A}\mathrm{m}}\cdot {e}^{+{\lambda}_{\mathrm{Am}}\cdot t}}{\left(1-{e}^{-{\lambda}_{\mathrm{Pu}}\cdot t}\right)} $$

where:

*A*_Pu0_: ^241^Pu activity in the time of sampling
30.11409: Constant value (*λ*_Pu_/*λ*_Am_)
$$ {A}_{{}^{241}\mathrm{A}\mathrm{m}} $$
^241^Am activity increment measured after 5–10 years
*λ*_Pu_: 0.048303 year^−1^ (counted for 14.35 years half-life time)
*λ*_Am_: 0.001604 year^−1^ (counted for 432.2 years half-life time)
*t*: Time from sampling to ^241^Am measurement (5–10 years)

The accuracy and the precision of the radiochemical methods of plutonium analysis were satisfactory (less than 7 %) and estimated by analysis of IAEA standard materials (IAEA-367, IAEA-384). The plutonium chemical yield varied from 60 to 90 %. The results of ^241^Pu activity concentration in analyzed samples are given with their 2σ SD confidence intervals. Statistic tests and small amount of samples analysis showed there was non-normal distribution of the data and the results showed statistically significant differences. All statistical procedures were based on non-parametric tests, Spearman’s correlation rank, and cluster analysis (Mazerski 2009).

## Study organisms

### Tufted duck (*A. fuligula*) (L., 1758)

The tufted duck is a medium-size duck that feeds mainly by diving (Dudziński 1998). These birds breed widely on Palearctic, from Iceland and Great Britain to the Kamchatka Peninsula (Kruszewicz 2005). All are migratory in most of their range and winter in the milder south and west of Europe, southern Asia; their migration starts in September (Żmudziński 1990; Kruszewicz 2005). These ducks feed on mixed vegetable/animal food, mainly mollusks which contribute to 92 % of the diet during winter and 60 % during spring; plants contribute less than 20 % of the diet (Kruszewicz 2005).

### Common eider (*S. mollissima*) (L., 1758)

The common eider is a large sea-duck that lives over the northern coasts of Europe, North America, and eastern Siberia. It breeds in the Arctic and some northern temperate regions, but winters over warmer areas and in large numbers (about 1 million individuals) and winters at the southern Baltic Sea, where they stay from September to April (Kruszewicz 2005). These ducks feed on invertebrates, mainly mollusks and crustaceans (Barrett et al. 2002).

### Long-tailed duck (*C. hyemalis*) (L., 1758)

The long-tailed duck is a medium-size duck that lives at sea coasts and in large mountain lakes in the North Atlantic region, Alaska, northern Canada, northern Europe, and Russia. They are migratory and the most important wintering area is the Baltic Sea, where a total of about 4.5 million gather from November to June (Kruszewicz 2005). They feed on bivalves, crustaceans, insects, and other small animals, i.e., small fish (Żmudziński 1990).

### Velvet scoter (*M. fusca*) (L., 1758)

The velvet scoter is a large diving migratory sea-duck which breeds over the far north area of Europe and Asia; the most of the European and Siberian population winters at the Baltic Sea from December to March (Hudek 1990; Tomiałojć and Stawarczyk 2003). These ducks feed on bivalves and small crustaceans, swallowed with the shell, and such diet is supplemented in crayfish, small fish, and insects (Hudek 1990).

### Eurasian coot (*F. atra*) (L., 1758)

The Eurasian coot occurs and breeds in middle and southern Europe, middle Asia, Australia, and north-eastern Africa. The migration starts in August with its maximum in October (Kruszewicz 2005). Coots are omnivorous birds; however, the diet depends on the food available. The main components are plant sprouts, grass and its seeds, as well as algae. Also, small vertebrates, fish, and frog spawn as well as bivalves appear (Hudek 1990; Kruszewicz 2005).

### Great cormorant (*P. carbo*) (L., 1758)

The great cormorant is a big water bird, mostly migratory, which lives in large continental areas, excluding South America and Antarctic, and among them, five subspecies can be observed. In Poland, the continental subspecies appears that winters at the Baltic Sea coast (Kruszewicz 2005). Great cormorants’ diet is composed of fish only, and they hunt on the bottom species mainly (Barrett et al. 2002).

### Razorbill (*A. torda*) (L., 1758)

The razorbill is a medium-size bird from Alcidae family that lives mainly on the northern sea islands and breeds across whole northern Atlantic Ocean, while in Europe from Scandinavia to France. It is completely connected to the sea where it finds food. It is mainly a nonmigratory bird; however, some migratory individuals can be observed in the western Baltic Sea from January to August (Hudek 1990). Razorbills’ diet is composed of small fish, mainly sprat, less often eel and gadids (Barrett et al. 2002).

### Black guillemot (*C. grylle*) (L., 1758)

The black guillemot is a pigeon-size bird from Alcidae family that breeds in northern Europe, Asia, and North America. Most of them are nonmigratory. They winter at the northern coast of the Atlantic Ocean and the Baltic Sea (Hudek 1990). The diet of the black guillemot is composed of fish mainly, sometimes of mollusks, crustaceans, and worms. Those from the Barents Sea feed on fish (60 %) and invertebrates (40 %) while from the Norwegian Sea on fish only (Żmudziński 1990; Barrett et al. 2002).

### Common guillemot (*U. aalge*) (Pont., 1763)

The common guillemot breeds in northern Europe, Asia, and North America and are mainly nonmigratory (Kruszewicz 2005). It is a fish-eating bird, mainly pelagic; however, some parts of the diet are crayfish, marine worms, crustaceans, mollusks, and polychaetes. The diet depends on the region they live: at the Kattegat dominate herrings and round gobies, at the Skagerrak—sprats and herrings, while in the central Baltic—sprats (Barrett et al. 2002).

### Red-throated diver (*G. stellata*) (Pont., 1763)

The red-throated diver is a duck-size bird that breeds primarily in the Arctic regions of northern Eurasia and North America, while winters in northern coastal waters, very often at the Baltic and the North Sea, as well as the Atlantic Ocean. The main food is fish with addition of spawn, crustaceans, mollusks, and insects (Kruszewicz 2005).

## Results and discussion

All results of ^241^Pu concentrations in analyzed seabirds are presented in Table 1. The obtained results indicated that the plutonium was non-uniformly distributed in organs and tissues of analyzed seabirds. Generally, the highest plutonium concentrations were found in the digestion organs and feathers, next in skeleton, and the lowest in muscles. Among analyzed birds, the highest ^241^Pu concentrations were found in viscera, its activities in the digestive organs ranged from 9.7 ± 2.5 mBq kg^−1^ wet weight (ww; 13.0 % of total ^241^Pu) in great cormorant to 228 ± 39 mBq kg^−1^ ww (79.6 % of total ^241^Pu) in velvet scoter (Table 1). High ^241^Pu concentrations were also found in liver, ranging from 21 ± 4 mBq kg^−1^ ww in velvet scoter (2.2 % of total ^241^Pu) to 159 ± 31 mBq kg^−1^ ww in Ttufted duck, and feathers ranged from 15 ± 4 mBq kg^−1^ ww in great cormorant (11.6 % of total ^241^Pu) to 132 ± 59 mBq kg^−1^ ww (34.2 % of total ^241^Pu) in common eider (Table 1). But the median value of ^241^Pu concentration in liver (60 mBq kg^−1^ ww) was higher in comparison with the median of its concentration in viscera and feathers (both 46 mBq kg^−1^ ww). The important observation is the liver, contributing a maximum of 6.5 % of the total body weight and feathers contributing less than 15 % of the total body weight while skeleton is part of the 24.7 to 28.7 % of the total body weight. Similar situation was observed in previously measured ^239+240^Pu when its highest concentrations were noticed in skeleton and feathers (Strumińska-Parulska et al. 2011). The lowest ^241^Pu concentrations were calculated for muscles (the median value of ^241^Pu concentration was 11 mBqa kg^−1^ ww) and ranged from 2.0 ± 0.6 mBqa kg^−1^ ww in velvet scoter to 30 ± 10 mBqa kg^−1^ ww in long-tailed duck. Also, the lowest values of ^239+240^Pu concentrations were observed in muscles (Strumińska-Parulska et al. 2011). According to all analyzed birds, the average concentration of ^241^Pu in the whole body ranged from 8.8 ± 0.4 mBqa kg^−1^ ww in great cormorant to 254 ± 46 mBqa kg^−1^ ww in velvet scoter (Table 1). The ^239+240^Pu and ^241^Pu were non-uniformly distributed in analyzed birds’ organs. The highest amounts were located in skeleton, which ranged from 9.3 % in great cormorant to 42.3 % in common eider of the total ^241^Pu content and viscera ranging from 9.6 % in common guillemot to 79.6 % in velvet scoter (Table 1). It was very difficult to compare these results from that of other researches, since there was no such detailed data on ^241^Pu accumulation. However, trying to compare with other radionuclides, these data agreed with our previous results for ^239 + 240^Pu concentrations and Lowe’s (1991) research as well where its highest concentrations in birds were noticed in viscera and the lowest in muscles (Strumińska-Parulska et al. 2011).

**Table 1:** Plutonium ^241^Pu in organs and tissues of analyzed seabirds

Organ/tissue	Sample		Average ^241^Pu concentration (mBq kg^−1^, ww)	Part of total ^239+240^Pu (%)	^241^Pu/^239+240^Pu activity ratio	BCF
	Wet weight (g)	Contribution (%)				
Great cormorant (*Phalacrocorax carbo*; *n* = 1)						
Liver	110	3.2	59 ± 9	21.6	27 ± 5	23 ± 4
Muscle	1075	31.3	8.5 ± 1.8	30.3	45 ± 15	3.3 ± 0.7
Feathers	239	7.0	15 ± 4	11.6	40 ± 16	5.7 ± 1.6
Skeleton	985	28.7	2.9 ± 0.7	9.3	57 ± 37	1.1 ± 0.3
Skin	624	18.1	6.8 ± 2.6	14.2	53 ± 23	2.7 ± 1.0
Viscera	404	11.7	9.7 ± 2.5	13.0	39 ± 13	3.8 ± 1.0
Whole body	3437	100.0	8.8 ± 0.4	100.0	40 ± 18	3.4 ± 0.1
Eurasian coot (*Fulica atra*; *n* = 1)						
Liver	–	–	–	–	–	–
Muscle	183	24.9	20 ± 4	25.5	58 ± 22	7.6 ± 1.5
Feathers	112	15.2	23 ± 6	18.4	44 ± 21	9.0 ± 2.3
Skeleton	190	25.9	19 ± 5	26.0	46 ± 19	7.5 ± 1.8
Skin	64	8.7	23 ± 6	10.4	44 ± 22	8.9 ± 2.3
Viscera	186	25.3	15 ± 5	19.6	7 ± 3	5.8 ± 1.9
Whole body (no liver)	735	–	19 ± 1	100.0	23 ± 11	7.4 ± 0.4
Razorbill (*Alca torda*; *n* = 9)						
Liver	40	4.6	73 ± 14	7.1	57 ± 22	28 ± 5
Muscle	205	23.4	10 ± 3	5.3	13 ± 4	4.1 ± 1.2
Feathers	114	13.0	55 ± 9	15.3	20 ± 4	21 ± 3
Skeleton	239	27.2	53 ± 13	31.4	34 ± 11	21 ± 5
Skin	187	21.3	64 ± 17	29.4	47 ± 17	25 ± 7
Viscera	92	10.5	50 ± 12	11.4	45 ± 16	20 ± 5
Whole body	877	100.0	46 ± 2	100.0	32 ± 16	18 ± 1
Tufted duck (*Aythya fuligula*; *n* = 2)						
Liver	49	5.0	159 ± 31	–	56 ± 24	62 ± 12
Muscle	211	21.4	12 ± 3	–	49 ± 20	4.6 ± 1.0
Feathers	92	9.3	51 ± 14	–	58 ± 19	20 ± 5
Skeleton	244	24.7	–	–	–	–
Skin	193	19.6	22 ± 4	–	57 ± 15	8.6 ± 1.5
Viscera	197	20.0	59 ± 9	–	35 ± 7	23 ± 4
Whole body	986	100.0	–	–	–	–
Common eider (*Somateria mollissima*; *n* = 13)						
Liver	117	4.7	27 ± 5	3.0	56 ± 22	10 ± 2
Muscle	593	23.6	10 ± 3	5.7	21 ± 8	3.9 ± 1.1
Feathers	272	10.8	132 ± 59	34.2	46 ± 25	51 ± 23
Skeleton	637	25.4	70 ± 15	42.3	37 ± 11	27 ± 6
Skin	436	17.4	–	–	–	–
Viscera	455	18.1	34 ± 5	14.8	28 ± 5	13 ± 2
Whole body	2510	100.0	50 ± 3	100.0	44 ± 25	20 ± 1
Long-tailed duck (*Clangula hyemalis*; *n* = 13)						
Liver	45	5.0	75 ± 15	6.4	50 ± 27	29 ± 6
Muscle	204	22.9	30 ± 10	11.7	38 ± 29	12 ± 4
Feathers	107	12.0	68 ± 16	13.7	50 ± 17	26 ± 6
Skeleton	252	28.2	60 ± 15	28.6	14 ± 4	23 ± 6
Skin	126	14.1	75 ± 20	17.9	50 ± 17	29 ± 8
Viscera	159	17.8	72 ± 12	21.7	50 ± 10	28 ± 5
Whole body	893	100.0	59 ± 2	100.0	28 ± 13	23 ± 1
Velvet scoter (*Melanitta fusca*; *n* = 12)						
Liver	85	4.7	21 ± 4	2.2	33 ± 10	8.4 ± 1.6
Muscle	450	24.9	2.0 ± 0.6	1.1	1 ± 0.4	0.8 ± 0.2
Feathers	197	10.9	–	–	–	–
Skeleton	483	26.7	22 ± 3	12.9	37 ± 7	8.5 ± 1.3
Skin	309	17.0	11 ± 5	4.2	14 ± 7	4.3 ± 1.9
Viscera	286	15.8	228 ± 39	79.6	57 ± 11	89 ± 15
Whole body	1810	100.0	254 ± 46	100.0	–	99 ± 18
Black guillemot (*Cepphus gryle*; *n* = 2)						
Liver	39	6.5	92 ± 19	15.8	54 ± 22	36 ± 7
Muscle	133	22.2	23 ± 5	13.6	58 ± 22	9.0 ± 1.8
Feathers	80	13.4	43 ± 8	15.3	13 ± 4	17 ± 3
Skeleton	165	27.5	19 ± 3	13.9	48 ± 23	7.4 ± 1.1
Skin	110	18.4	14 ± 3	6.8	30 ± 10	5.5 ± 1.0
Viscera	72	12.0	109 ± 19	34.6	26 ± 6	42 ± 8
Whole body	599	100.0	38 ± 2	100,0	28 ± 13	15 ± 1
Red-throated diver (*Gavia stellata*; *n* = 7)						
Liver	90	4.6	38 ± 7	4.7	48 ± 16	15 ± 3
Muscle	544	27.7	23 ± 5	16.8	47 ± 15	8.8 ± 2.0
Feathers	250	12.8	43 ± 8	14.6	24 ± 5	17 ± 3
Skeleton	489	24.9	65 ± 19	43.6	39 ± 17	25 ± 7
Skin	350	17.8	14 ± 3	6.5	12 ± 4	5.3 ± 1.3
Viscera	240	12.2	43 ± 11	13.9	35 ± 11	17 ± 4
Whole body	1963	100.0	37 ± 2	100.0	32 ± 17	15 ± 1
Common guillemot (*Uria aalge*; *n* = 2)						
Liver	44	4.5	61 ± 15	12.7	45 ± 21	24 ± 6
Muscle	301	30.6	10 ± 2	14.7	45 ± 33	4.1 ± 0.9
Feathers	100	10.2	48 ± 9	22.7	39 ± 10	19 ± 3
Skeleton	280	28.4	18 ± 4	24.2	48 ± 36	7.1 ± 1.5
Skin	191	19.4	18 ± 4	16.1	60 ± 37	7.0 ± 1.7
Viscera	68	6.9	30 ± 6	9.6	44 ± 18	12 ± 2
Whole body	984	100.0	22 ± 1	100.0	46 ± 22	8.4 ± 0.3

On the basis of ^241^Pu/^239+240^Pu activity ratio, there was an attempt to indicate the main sources of ^241^Pu in analyzed bird samples. Among all analyzed species, the highest value of ^241^Pu/^239+240^Pu activity ratios were found in the body of common guillemot (46 ± 22), common eider (44 ± 25), and great cormorant (40 ± 18). However, looking at the significant uncertainties, even as high as 60 %, it was not possible to clearly indicate the main source of plutonium in analyzed seabirds. We could only suppose that the global atmospheric fallout had significant impact on total plutonium contribution in analyzed bird samples.

The normalized partition factor (PF) could be used as a good coefficient to describe the distribution of the radionuclides in analyzed birds’ organisms (Table 2) (Mietelski 2003). The PF for analyzed organs was defined as a ratio of ^241^Pu percentage contribution in analyzed organ to the percentage mass contribution of the organ (Mietelski 2003). The PF values in analyzed tissues and organs reflected the radionuclide distribution in the seabirds organisms and PF >1 indicated radionuclide accumulation in organ or tissue of the analyzed organism. On the basis of obtained PF values, we could notice that three organs accumulated ^241^Pu the most: liver, feathers, and viscera; however, the liver seemed to be the most important organ in plutonium accumulation with its highest median value of 2. The median values for feathers and viscera were both calculated at 1.2. Among these organs, the highest PF values were calculated for livers of great cormorant (6.7), further common guillemot (2.8), and black guillemot (2.4); viscera of black guillemot (2.9), common eider (1.4), and long-tailed duck (1.2), and feathers of common eider (2.2) and great cormorant (1.7) (Table 2). According to Spearman’s rank correlation analysis, high coefficient was calculated between ^241^Pu concentrations in skin and feathers (0.83) (Table 3). Our previous studies on ^239 + 240^Pu showed that only a part of plutonium is built in feather structure while the rest could be on the feathers as a result of preening with preen oil from uropygial glands or adsorbed from the atmosphere (Strumińska-Parulska et al. 2011). Although the waterproofing effect was not primarily by the uropygials—feather are hydrophobic—but by applying an electrostatic charge to the oiled feather through the mechanical action of preening using uropygial gland, plutonium could be adsorbed on the feathers (Furness and Camphuysen 1997; Pilastro et al. 1993; Møller et al. 2010). Generally, the highest ^241^Pu concentrations in the whole body were observed in wintering seabirds (velvet scoter, long-tailed duck, common eider) and further in migratory seabirds (razorbill, red-throated diver, common guillemot); migratory birds had the highest ^241^Pu concentrations in feathers as well. Seabirds that permanently reside at the southern Baltic Sea were characterized by the highest plutonium concentrations in liver (great cormorant, tufted duck). Similar situation was observed in the previous studies on ^239 + 240^Pu in seabirds from the southern Baltic Sea (Strumińska-Parulska et al. 2011).

**Table 2:** The PF values (normalized partition factor) for ^241^Pu in organs and tissues of analyzed seabirds

Organ/tissue	Species					
	Great cormorant	Razorbill	Common eider	Long-tailed duck	Black guillemot	Red-throated diver
Liver	6.7	1.6	2.8	1.3	2.4	1.0
Muscle	1.0	0.2	0.5	0.5	0.6	0.6
Feathers	1.7	1.2	2.2	1.1	1.1	1.1
Skeleton	0.3	1.2	0.9	1.0	0.5	1.7
Skin	0.8	1.4	0.8	1.3	0.4	0.4
Viscera	1.1	1.1	1.4	1.2	2.9	1.1

**Table 3:** Spearman’s rank correlation coefficient of ^241^Pu concentrations between analyzed seabirds’ organs and tissues

Organ/tissues	Liver	Muscle	Feathers	Skeleton	Skin	Viscus
Liver	1.00					
Muscle	0.45	1.00				
Feathers	0.45	0.32	1.00			
Skeleton	0.15	0.22	0.56	1.00		
Skin	0.22	0.43	0.83	0.47	1.00	
Viscus	0.38	0.25	0.09	0.33	0.08	1.00

Our data obtained for analyzed seabirds were in good agreement with plutonium isotopes distribution in marine animals. In fish, the highest ^241^Pu concentrations were observed in alimentary system, lower in skeleton, and the lowest in muscles (Skwarzec 1995; Skwarzec et al. 2001; Strumińska-Parulska and Skwarzec 2013). The main route of plutonium intake by humans is aerosols inhalation, but marine animals (zoobenthos and fish) assimilate it from food (ICRP 1986; Skwarzec 1995; Strumińska-Parulska et al. 2011; Strumińska-Parulska and Skwarzec 2013). It was in agreement with Krivolutski’s observation as well. He and his collaborators noticed that radionuclides entered the body via the food chains of the radioactive ecosystem (Krivolutsky et al. 1999). The concentrations of ^241^Pu in seabirds were much lower in comparison with fish from the southern Baltic Sea (average value of ^241^Pu concentration in fish was 441 ± 20 mBq kg^−1^ ww) as well as in comparison with phytobenthos, crustaceans, and bivalves (mean values of ^241^Pu concentrations were 457 ± 91, 2337 ± 318, and 913 ± 64 mBq kg^−1^ ww, respectively) (Strumińska and Skwarzec 2006; Strumińska-Parulska and Skwarzec 2013). On the basis of the average ^241^Pu concentrations in the southern Baltic Sea biocenosis components, the plutonium content in marine organisms increased as: seabirds < fish < phytobenthos < phytoplankton < zooplankton < zoobenthos; similarly to ^239 + 240^Pu and other radionuclides (Strumińska-Parulska et al. 2011; Howard et al. 2013).

**Fig. 2:**
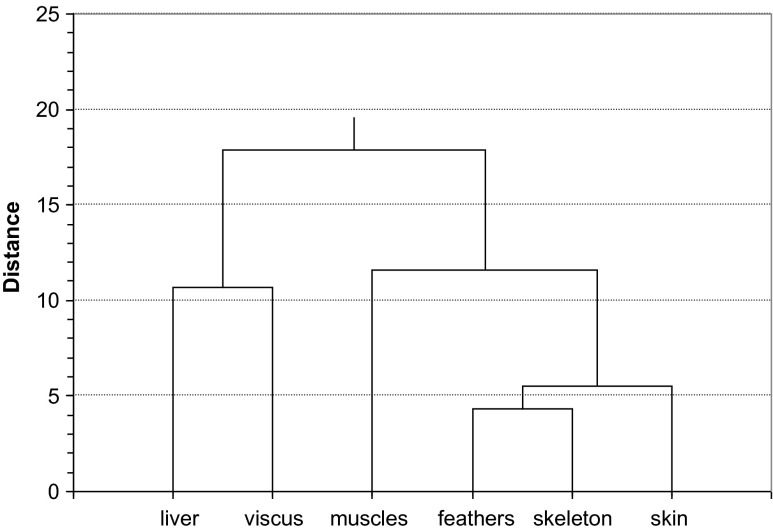
Cluster analysis (Ward’s method) of ^241^Pu concentrations in the analyzed seabirds’ organs and tissues

**Fig. 3:**
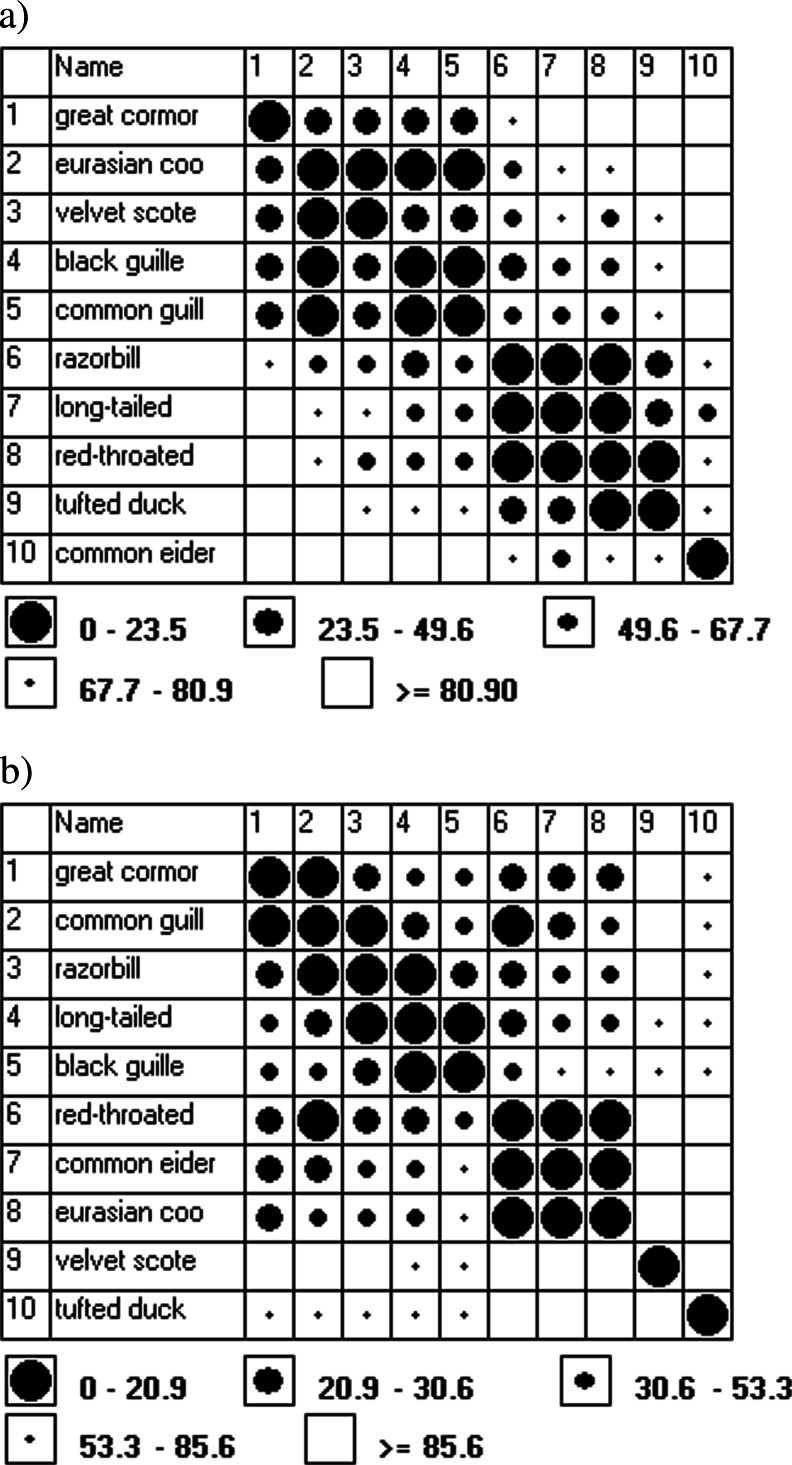
Percent differences between ^241^Pu concentrations in analyzed birds as ordered Euclidean Czekanowski’s diagrams on the basis of standardized data depending on **a** skeleton and feathers and **b** liver and viscera

Statistical analysis of ^241^Pu concentrations values in analyzed seabirds based on statistical test and small amount of samples showed non-normal distribution of the data. Further chemometric analysis was based on non-parametric tests, Spearman's rank correlation coefficient and cluster analysis (CA). Using cluster analysis (Ward’s method), we could separate one main sub-group with the lowest differences: feather-skeleton connected to skin sub-group (Fig. 2). Also, liver-viscus sub-group was noticed; however, the similarities were not as significant as feathers-skeleton sub-group. On the basis of chosen groups, we were looking for some differences between analyzed seabirds. Searching for further information, the blocks of analyzed seabirds as the percentage differences between individual bird species on the basis of obtained sub-groups were calculated and presented on the basis of ordered Euclidean Czekanowski’s diagrams (Fig. 3). These diagrams, which described an index of similarity between two samples, were often used as universal statistical classification with some elements of correspondence analysis (Mazerski 2009). According to feather-skeleton sub-group (Fig. 3a), we could notice the lowest differences based on migration and further on birds’ diet. The lowest differences were found between wintering birds and birds feeding on mixed food (velvet scoter-black guillemot and Eurasian coot-black guillemot-common guillemot) as well as migratory birds feeding on fish (razorbill-red-throated diver-tufted duck). On the basis of slight similarities of each bird in the case of liver and viscera at Czekanowski’s diagram (Fig. 3b), we could observe that the plutonium bioaccumulation in these organs depended mainly on the birds’ diet: block of great cormorant-razorbill and long-tailed duck-black guillemot-common guillemot feeding of fish and mollusks, respectively.

**Fig. 4:**
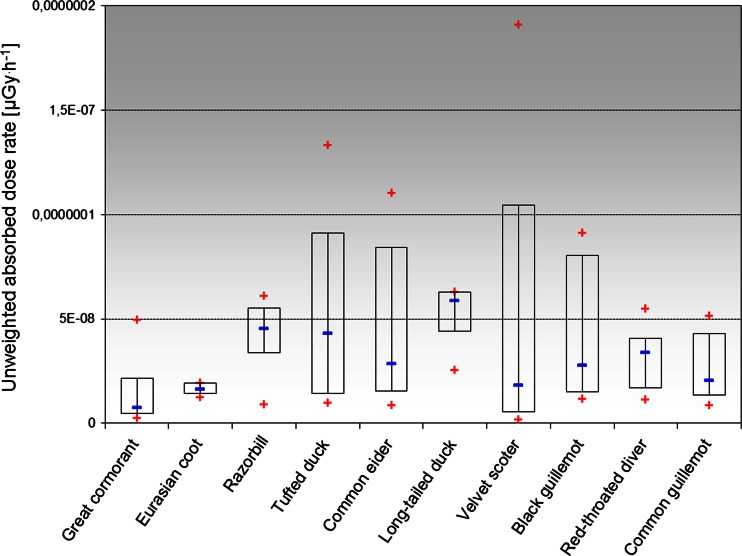
The mean, minimum, and maximum values of unweighted absorbed dose rate for analyzed birds’ organs and tissues from ^241^Pu accumulated

Toxic substances like metals, sulfides, radionuclides, and reactive oxygen species cause DNA damage in organisms (Pruski and Dixon 2003). Regarding radionuclides, toxicity is related to the amount of energy deposited in an organ over time. Internal emitters keep emitting their radiation inside the body. Therefore, the cumulative effect of multiple small doses of radiation from internal emitters could be even more dramatic, depending on the half life, metabolic pathways, and other properties of the particular radioactive particle. Chemical changes in DNA are basic for radiation damage and the double-strand break (DSB) is the most important type of DNA damage in relation to ionizing radiation (Schwartz 2007). Of course, the damage from huge single dose may be greater than the same cumulative dose from many small exposures. But the smaller doses can still add up. Using the dose conversion factors given by UNSCEAR (2000), the unweighted absorbed dose rates (μGy h^−1^) for birds were calculated, and their values were presented in Table 4. The values of mean, minimum, and maximum of unweighted absorbed dose rate for analyzed birds organs and tissues from ^241^Pu accumulated were given on Fig. 4. The results showed the biggest unweighted internal dose rates were calculated for velvet scooter (2.13 × 10^−7^ μGy h^−1^) while the lowest for great cormorant (7.33 × 10^−9^ μGy h^−1^) (Table 4), but the calculations showed the calculated doses were very low, comprising with other isotopes or species, e.g., ^210^Po or fish (Skwarzec 1995; Strumińska-Parulska and Skwarzec 2013). On the basis of present knowledge and radiation dose limits, it could be concluded that the calculated doses had no significant influence on the organisms. However, the validity of using dose–response model is controversial because evidence accumulated over the past has indicated that living organisms respond differently to low dose/low-dose-rate radiation than they do to high dose/high-dose-rate radiation. There are accumulated findings which cannot be explained by the classical “target theory” of radiation biology (Matsumoto et al. 2007).

**Table 4:** Unweighted absorbed dose rate for analyzed birds whole body from ^241^Pu accumulated

Bird species	Unweighted absorbed dose rate (μGy h^−1^)
Great cormorant	7.33 × 10^−9^ ± 2.98 × 10^−10^
Eurasian coot	1.60 × 10^−8^ ± 8.12 × 10^−10^
Razorbill	3.88 × 10^−8^ ± 1.51 × 10^−9^
Common eider	4.23 × 10^−8^ ± 2.46 × 10^−9^
Long-tailed duck	4.96 × 10^−8^ ± 1.99 × 10^−9^
Velvet scoter	2.13 × 10^−7^ ± 3.83 × 10^−8^
Black guillemot	3.17 × 10^−8^ ± 9.79 × 10^−10^
Red-throated diver	3.14 × 10^−8^ ± 1.31 × 10^−9^
Common guillemot	1.81 × 10^−8^ ± 6.51 × 10^−10^

## Conclusions

The bioaccumulation of plutonium in seabirds depended not only on its concentration in the food but also in the environment. Seabirds are typical multi-habitat animals, and radionuclides can come from water and air. Some thought seabirds, especially their feathers, could be a good indicator for radiological biomonitoring and radioactive pollution. However, data showed that seabirds, even their feathers, were not very useful in plutonium monitoring because its accumulation efficiency was quite low and decreased within the food chain. Obtained data indicated that seabirds were an important chain in all plutonium radionuclide environmental migration and showed that its content decreased as liver > viscera > feathers > skeleton > skin > muscles. Used chemometric analysis (Spearman’s correlation rank, CA) allowed for conclusion that the main sources of plutonium in seabirds were food and behavior (living habits). This observation could be confirmed by plutonium distribution in organs and tissues of analyzed seabirds compared with their diet.

Analysis of the internal radiation doses coming from ^241^Pu accumulated in seabirds showed these doses were very low and had no significant influence on the organisms.
